# Manipulating DNA repair and the DNA damage response to improve cancer therapy

**DOI:** 10.1093/narcan/zcag006

**Published:** 2026-02-18

**Authors:** Li Lan, Elise Fouquerel

**Affiliations:** Departments of Molecular Genetics and Microbiology, School of Medicine, Duke University, 213 Research Dr, Room 424, Durham, NC 27710, United States; UPMC Hillman Cancer Center, Department of Pharmacology and Chemical Biology, University of Pittsburgh Cancer Institute, Pittsburgh, PA 15213, United States

*NAR Cancer* is pleased to present a thematic collection of 17 articles focused on how progress in understanding the DNA damage response (DDR) has reshaped modern cancer therapy. The impetus for this collection came from the journal’s 5th Anniversary Symposium, held in cooperation with the popular *Social DNAing* webinar series. Some of the articles in the collection were contributed by symposium speakers, whereas others came in response to a call for papers. The collection includes Critical Reviews and Perspectives, Short Reviews, and standard papers. Links to relevant, previously published articles are also included on the collection home page (https://academic.oup.com/narcancer/pages/manipulating-dna-repair).

The clinical success of PARP inhibitors established synthetic lethality as a viable therapeutic strategy, demonstrating that DDR-targeting drugs can selectively kill repair-defective cancers. Yet, resistance, pathway rewiring, and the activation of backup repair routes remain major challenges. As the field moves beyond the PARP inhibition paradigm, attention has expanded to the broader signaling landscape and additional therapeutic target options, including repair helicases, DNA/RNA structure-specific repair, posttranslational regulation of repair complexes, and emerging technologies such as CRISPR-based screening, optogenetic modulation, and machine-learning-enabled biomarker quantification. This collection aims at capturing the momentum in the DNA repair field that combines basic biology with technical and clinical innovations.

DNA repair and maintenance pathways, including homologous recombination (HR), base excision repair (BER), and mismatch repair (MMR), operate within an interconnected network that responds to replication stress, transcription, oxidative damage, chromatin context, RNA modifications, and metabolic state. Cancer cells often harbor defects in these canonical pathways while exploiting compensatory mechanisms such as polymerase θ-mediated end joining, R-loop processing, and telomerase and ALT-mediated telomere maintenance. These altered dependencies create rich opportunities for therapeutic exploitation, and the articles in this thematic collection highlight advances across mechanistic biology, innovative technologies, and translational applications.

## Homologous recombination, transcription-coupled repair, and HR-directed therapeutics

Several articles explore the regulation of HR and strategies to target HR-defective tumors. “Automated machine learning profiling with MAP-HR for quantifying homologous recombination foci in patient samples” introduces a machine-learning approach for quantifying RAD51 and other HR foci in patient tissues, enabling functional assessment of HR status to guide therapeutic decisions [1]. The crosstalk between kinase signaling and transcription-coupled repair is illuminated in “ABL1 kinase-mediated tyrosine phosphorylation of SYCP2 contributes to transcription-coupled homologous recombination and platinum resistance in ovarian cancer,” which demonstrates that ABL1-driven phosphorylation of SYCP2 promotes HR at actively transcribed regions [2].

A complementary synthetic lethal vulnerability is described in “Polymerase θ—what does it see, and why does it matter for cancer therapy?,” which outlines how HR-deficient tumors rely heavily on Pol θ-mediated end joining, making Pol θ inhibition a promising therapeutic strategy [3]. Mechanisms that undermine these strategies are discussed in “PARP inhibitor resistance in IDH1-mutant cancers due to loss of end protection factors, 53BP1 and Rev7,” which identifies metabolic and repair rewiring processes that confer resistance and suggests avenues for rational combination therapies [4].

## Base excision repair, oxidative damage, and genome maintenance

The BER pathway and oxidative DDRs emerge as central determinants of treatment response. Accordingly, there has been a significant effort to improve oxidative stress-generating tools. These tools can be used to better understand how specific regions of the genome are impacted by oxidative stress and for mapping repair dependencies. They are reviewed in “Genetic approaches for targeted oxidative stress” [5]. The contextual sensitivity of oxidative lesion repair is further detailed in “Base excision repair within structure-forming repeats sequences and its impact on cancer and other diseases,” which bring insights into this emerging aspect of the BER pathway [6].

The BER pathway is orchestrated by a plethora of enzymes that coordinate efficient repair. Among these enzymes are the DNA glycosylases, whose diversity allows the removal of many different base lesions and whose activities can expand beyond their canonical functions. The diversity and evolving functions of BER glycosylases are exemplified in “What’s in a name? Rethinking SMUG1 in genome maintenance,” which discusses SMUG1’s role beyond classical uracil excision [7]. Distinct functions are also presented for the BER nucleases, APE1 and APE2, in “Contrasting roles of APE1 and APE2 in genome maintenance, cancer development, and therapeutic targeting,” which highlights how these enzymes shape mutational landscapes and therapeutic vulnerability [8]. Finally, “Replication-associated BER repair/SSB repair regulates PARG inhibitor response via the PRMT1/PRMT5/ATR axis” shows how proteins involved in the processing of BER repair intermediates influence sensitivity to PARP and PARG inhibitors via replication stress signaling, refining the framework of BER–PARP synthetic lethality [9].

## Structure-specific repair, noncanonical DNA/RNA structures, and mitochondrial stress responses

Several articles highlight the therapeutic relevance of noncanonical DNA and RNA structures. “G-quadruplex ligand RHPS4 compromises cellular radioresistance by inhibiting the mitochondrial adaptive response induced by ionizing irradiation” demonstrates how stabilizing G-quadruplexes disrupts mitochondrial stress signaling to reduce radioresistance [10]. Epitranscriptomic regulation of genome stability is presented in “m^6^A modifications in R-loop homeostasis: a potential target for cancer therapeutics,” which identifies m^6^A-dependent R-loop regulation as an emerging therapeutic axis [11]. Notably, ABL1–SYCP2 signaling, as described above, also operates at R-loop-associated regions, further connecting transcriptional activity to HR-directed repair [2].

Mismatch repair dysfunction arising from disrupted protein interactions is described in “Disruption of protein–protein interaction hotspots in the C-terminal domain of MLH1 confers mismatch repair deficiency,” illustrating how perturbation of MLH1 interfaces drives MMR failure at T/G mismatches and CAG extrusion and is associated with tumor phenotypes [12].

## DNA repair helicases, biomolecular condensates, and the ALT pathway: promising therapeutic targets

Enzymatic motors and spatial organization of DDR signaling are emerging areas of therapeutic interest. “DNA repair helicases: from mechanistic understanding to therapeutic implications” offers a comprehensive overview of helicase functions in replication fork maintenance, HR, and DNA damage tolerance, as well as their potential as drug targets [13]. Spatial and phase-separated dimensions of DDR signaling are explored in “Shining light on drug discovery: optogenetic screening for TopBP1 biomolecular condensate inhibitors,” which uses optogenetic control of TopBP1 condensates to identify small molecules capable of disrupting condensate-mediated signaling [14].

Telomeres represent a distinct noncanonical repair context in cancer. “The alternative lengthening of telomeres pathway through a DNA repair lens: mechanism and therapeutic opportunities” provides a detailed view of recombination-driven telomere maintenance and highlights ALT-specific factors as promising therapeutic targets [15].

## Toward emerging therapeutic strategies and novel targets

A broad pharmacological perspective is provided in “Targeting DNA repair mechanisms in cancer therapy: the role of small molecule DNA repair inhibitors,” which reviews inhibitors of PARP, ATR, DNA-PK, Pol θ, BER enzymes, and other DDR factors [16]. Finally, “Targeted CRISPR knockout screening identifies known and novel chemogenomic interactions between DNA damaging agents and DNA repair genes” maps and offers an opportunity to expand the diversity of DDR-targeted agents and their potential in precision oncology [17].

## Conclusion

Together, these studies provide a coherent framework for understanding how tumors repurpose DNA repair pathways and how these adaptations can be therapeutically targeted. By integrating mechanistic insights with innovative technologies—from machine learning and optogenetics to CRISPR screening and structure-specific repair mapping—this collection highlights the rapidly expanding opportunities to manipulate DDR pathways for clinical benefit. As DDR-targeted therapies continue to evolve, a deeper understanding of pathway interplay, noncanonical structures, and resistance mechanisms will be essential for designing durable, effective treatments that exploit the unique vulnerabilities of cancer genomes.
